# Herpetic whitlow in a child with AIDS: the importance of Tzanck test in the diagnosis^☆^^☆☆^

**DOI:** 10.1016/j.abd.2020.08.017

**Published:** 2021-05-18

**Authors:** Ricardo Barbosa Lima, Mariana de Almeida Pinto Borges, Luciana Ferreira de Araújo, Carlos José Martins

**Affiliations:** Universidade Federal do Estado do Rio de Janeiro, Rio de Janeiro, RJ, Brazil

**Keywords:** Cytology, Diagnosis, Fingers, Herpes Simplex, HIV

## Abstract

Herpetic whitlow is a viral infection of the fingers caused by the herpes simplex virus. The disease has a bimodal age distribution, affecting children under 10 years of age and young adults between 20 and 30 years old. It can be easily mistaken for panaritium or bacterial cellulitis. In patients with AIDS, atypical, chronic and recurrent ulcerated lesions occur. The Tzanck test allows a quick and low-cost diagnosis of herpes simplex virus infection. The authors report the case of a child with AIDS with painful finger ulcers in which the diagnosis was confirmed by the Tzanck test.

## Introduction

Herpetic whitlow (HW) is an acute viral infection of the fingers caused by herpes simplex virus (HSV) type 1 or 2.^1^ The disease has a bimodal age distribution, affecting children under 10 years and young adults between 20 and 30 years. HW is caused in children almost exclusively by HSV-1.^2^ It can easily be mistaken for bacterial panaritium or cellulitis due to the similarity of signs and symptoms.^1^, ^2^, ^3^, ^4^ In HIV-infected children, the HSV infection can cause atypical, chronic and recurrent ulcerated lesions, which can result in diagnostic errors.^5^ Moreover, HW may be the first indicator of an asymptomatic HIV infection.^1^, ^5^

Diagnostic tests include viral culture, the measurement of serum antibodies, the Tzanck test (cytology), and the screening for HSV-specific antigen in the lesion. The Tzanck tests or the antigen detection are indicated for a rapid diagnosis.^2^, ^3^, ^6^ The detection of the HSV DNA by real-time PCR (RT-PCR) is considered the diagnostic method of choice.^6^, ^7^ However, the Tzanck test provides a fast and low-cost diagnosis.^8^

## Case report

A six-year-old boy was admitted to the pediatric ward with diarrhea, anorexia, severe malnutrition, anemia, and painful ulcers in his fingers and on the face. The father reported the onset of finger lesions six months before, associated with the child's habit of biting his fingertips. The child had congenital HIV infection and made irregular use of antiretroviral therapy. The CD4 count was 5 cells/mm^3^ (2.07%), the CD8 count was 170 cells/mm^3^ (72.24%), and the CD4 / CD8 ratio was 0.03. The viral load was 34.578 copies/mL (log = 4.539).

**Figure 1:**
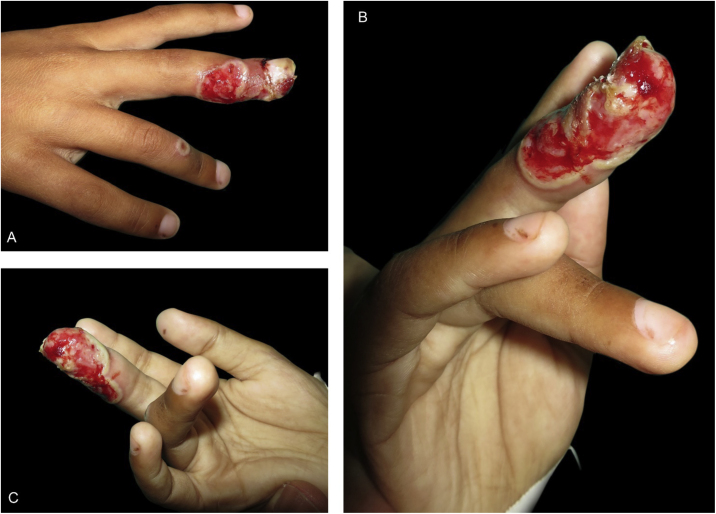
(A-B-C) Extensive ulceration with the border outlined by a whitish epidermal flap on the third right finger, with periungual involvement. On the fourth right finger an umbilicated vesicle can be observed.

**Figure 2:**
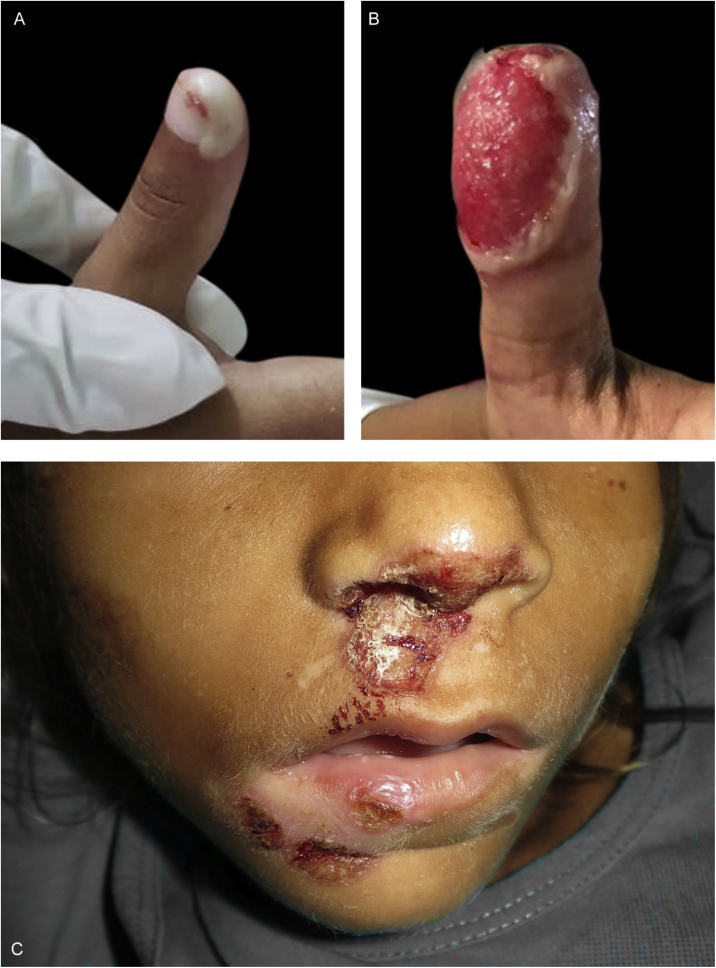
(A) Blister on the distal phalanx of the right thumb. (B) ulceration outlined by an epidermal flap on the distal phalanx of the left thumb. (C) crusted erosions and hypochromic areas on the nose and lips.

On physical examination, he had extensive ulcerations on the third right finger, with borders outlined by a whitish epithelial flap and periungual involvement. An umbilicated vesicle was seen on the fourth right finger, (Fig. 1). A blister was observed on the right thumb and the left thumb had an ulceration (Fig. 2A–B). His nose and lips showed erosions with crusts and hypochromic areas (Fig. 2C). He reported severe pain due to the finger lesions (Wong-Baker face pain rating scale = 5). Amoxicillin/clavulanate was started and dermatological assessment was requested, which raised the diagnostic hypothesis of HW and herpes simplex labialis.

**Figure 3:**
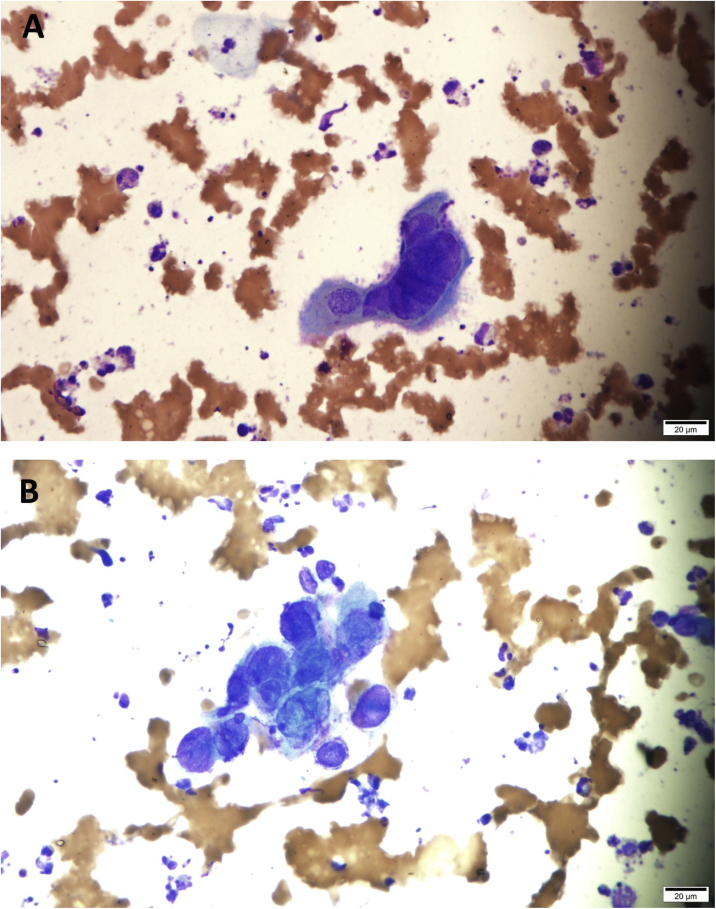
(A–B) multinucleated squamous cells with nuclear molding and ground-glass appearance compatible with HSV viral infection (Giemsa, ×400).

**Figure 4:**
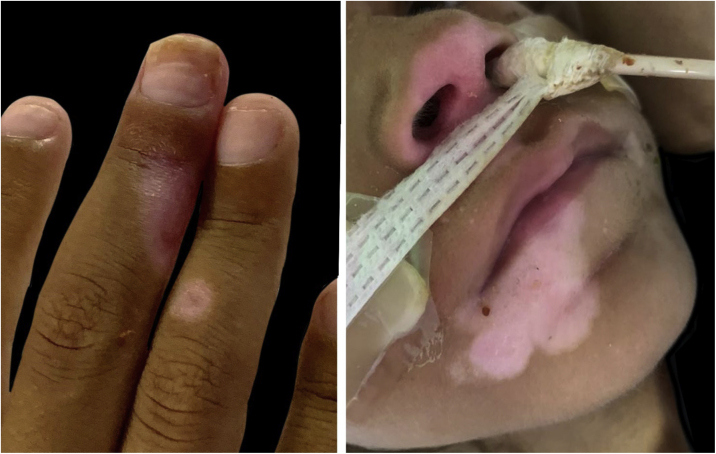
Residual hypochromic lesions after acyclovir therapy.

It was decided to collect material for a Tzanck smear and perform a biopsy for histopathological examination. The scraping of the ulcer base was performed; however, it was not possible to perform the biopsy due to the child's intolerance to pain. The cytological examination of the Giemsa-stained smear showed several multinucleated squamous cells with nuclear molding and ground-glass appearance compatible with HSV viral infection (Fig. 3) Treatment with acyclovir was introduced (500 mg/m^2^ every 8 hs/IV for 21 days) with rapid healing of the lesions (Fig. 4).

## Discussion

The natural evolution of HW is the complete resolution in three weeks. However, immunosuppressed patients are at risk for more frequent recurrences and chronic and severe HSV lesions, as observed in this patient.^1^, ^2^, ^5^ The involvement of more than one finger is unusual, but the coexistence of HSV lesions in the mouth and fingers, as in the present case, is a frequent finding.^9^, ^10^ A peculiarity of this patient was the habit of biting the fingertips, as described in other cases of HW in children with oral manifestations of the virus.^2^

The difficulty in establishing a diagnosis is frequently mentioned in the literature.^1^, ^2^, ^3^, ^4^ In the present case, the clinical aspect with painful ulcerations, vesicles, and blisters on the fingers, concomitantly with the labial lesions, in a child with AIDS, led to the diagnostic hypothesis of HSV infection.^3^

Aiming to rapidly confirm the diagnosis, a sample was obtained by scraping the ulcer base and the Tzanck test was performed, because RT-PCR was not available in the hospital where the study was done.^6^, ^8^ The biopsy was postponed due to the child’s pain; however, after a few hours, the cytological examination revealed the cytopathic alterations characteristic of HSV, which were sufficient to confirm the clinical diagnosis and initiate therapy with acyclovir IV.^8^

The Tzanck test, or smear, is a fast, easy to perform, low-cost test that showed to be very useful in the present case, especially in a child, because it does not require anesthesia.^2^, ^8^ The presence of multinucleated giant epithelial cells under optical microscopy confirms the diagnosis.^6^, ^7^, ^8^

In conclusion, the authors of this present study highlight that in the presence of chronic painful ulcerations in the fingers of children with AIDS, the diagnosis of HW should be considered and the Tzanck test should be performed as a useful method to confirm the diagnosis.

## Financial support

None declared.

## Authors’ contributions

Ricardo Barbosa Lima: Creation and overall writing of the manuscript taking the photographs and preparation of the figures; physician who guided the patient’s diagnosis and treatment.

Mariana de Almeida Pinto Borges: Participated in the preparation of the manuscript by collecting and organizing the data and review of the final version of the manuscript; pediatrician who carried out the patient’s clinical follow-up.

Luciana Ferreira de Araújo: Participated in the preparation of the manuscript through the analysis; description and photographs of the cytological examination and review of the final version of the manuscript; pathologist who performed the patient's cytological examination.

Carlos José Martins : Preparation and writing of the manuscript; critical review of the manuscript; physician who guided the collection and preparation of the patient's cytological examination.

## Conflicts of interest

None declared.
